# Feasibility of using health and wellbeing data for school planning: the SHINE pilot in Scotland

**DOI:** 10.1093/heapro/daac149

**Published:** 2022-11-28

**Authors:** Stephanie Chambers, Dawn Haughton, Judith Mabelis, Judith Brown, Jo Inchley

**Affiliations:** School of Social and Political Sciences, University of Glasgow, Glasgow, UK; MRC/CSO Social and Public Health Sciences Unit, University of Glasgow, Glasgow, UK; MRC/CSO Social and Public Health Sciences Unit, University of Glasgow, Glasgow, UK; MRC/CSO Social and Public Health Sciences Unit, University of Glasgow, Glasgow, UK; MRC/CSO Social and Public Health Sciences Unit, University of Glasgow, Glasgow, UK; MRC/CSO Social and Public Health Sciences Unit, University of Glasgow, Glasgow, UK

**Keywords:** health and wellbeing, schools, mental health, children and young people, whole-school approach

## Abstract

Child and adolescent mental health and wellbeing (MHWB) have received greater attention in recent years due to increases in mental ill health and reports of decreasing subjective wellbeing. The School Health and Wellbeing Improvement Research Network (SHINE) was established to create a national infrastructure to support Scottish schools to collect and use health and wellbeing (HWB) data to inform school improvement action planning. This study aimed to evaluate a pilot of SHINE’s provision of school-level HWB data reports from the Health Behaviour in School-aged Children survey and their impact on school action planning. Using a qualitative case study design, we collected data in four local authorities across Scotland via pupil and school staff focus groups (*n* = 23 groups), and from interviews with senior leaders, school SHINE Leads, other relevant school-level stakeholders, local authority (LA) HWB and data leads (*n* = 30 interviews). Data analysis was supported using Normalisation Process Theory as a guiding framework. Implementation was at an early stage. Participants indicated that the data reports were an accessible and valuable source of local information to support the improvement agenda. SHINE’s expertise supported the lack of research capacity and strengthened HWB data literacy skills in schools. At the point of interview, data reports had not been shared widely within the school community, but there was some limited use of the reports to inform action planning around HWB. Through close working and further engagement with schools, SHINE has the potential to support them to deliver national commitments to improving HWB.

## BACKGROUND

Young people’s mental health and wellbeing (MHWB) in the UK are worsening. A 2015 review (Collishaw, 2015) reported increases in the diagnoses of neurodevelopmental disorders (such as autism and ADHD) as well as affective disorders (such as depression and anxiety). Similarly, an international systematic review of adolescent mental health trends from the 1980s/90s until 2013 found that internalizing symptoms such as depression are increasing, particularly amongst girls (Bor *et al.*, 2014). In England, the proportion of school-aged children reporting a probable mental disorder increased from 11% in 2017 to 16% in 2020 (Vizard *et al.*, 2020). In Scotland, there has been a 22% increase in referrals to specialist mental health services between 2013 and 2018 (Audit Scotland, 2018). Furthermore, results from the Scottish Schools Adolescent Lifestyle and Substance Use Survey (SALSUS) show that since 2010, the proportion of young people in Scotland with reported mental health problems has continually risen, especially amongst adolescent girls (Scottish Government, 2020). Kim-Cohen *et al.* (2003) found that 75% of adults accessing mental health services had a diagnosable mental disorder before 18 years, highlighting the need for prevention and early intervention during childhood and the adolescent years. 

### Schools and health promotion

The Scottish Government’s Mental Health Strategy (2017a) focuses on prevention and early intervention. It identifies schools as key settings in which to implement prevention strategies including providing children and young people with access to emotional and mental wellbeing support, improved mental health training for those working with young people and implementation of evidence-based interventions. The Scottish Government’s approach to improving children and young people’s wellbeing is outlined in Getting it Right for Every Child (GIRFEC) (Scottish Government, 2021a) with eight key indicators which measure whether children are being supported to be safe, healthy, achieving, nurtured, active, respected, responsible and included. Within Scotland’s Curriculum for Excellence, HWB shares equal status with literacy and numeracy and is seen as the responsibility of all teaching and non-teaching school staff (Scottish Government, no date). The Scottish Government (2017b) recommends a data-driven approach to inform school decision-making and planning, particularly around health and wellbeing (HWB), and for the implementation and monitoring of evidence-based interventions. More recently, a Whole School Approach to MHWB has been launched (Scottish Government, 2021b) which provides a framework for local authorities and schools to embed support for children and young people’s MHWB across all aspects of the school environment.

Internationally, there has also been substantial focus on the ways in which schools can support children and young people’s HWB. The World Health Organisation has highlighted the important role of Health Promoting Schools (World Health Organisation, 2021), where the school environment, education and other school and community services cohesively provide opportunities to improve the health of children, families, communities and school staff. Previous intervention studies have shown that a Whole School Approach to MHWB can enhance children’s social and emotional development (Goldberg *et al.*, 2019). This broad approach may be more effective at improving health and behaviours than singular health interventions (Bond *et al.*, 2004) and has the potential to lead to more sustainable change (Svane *et al.*, 2019). There have been criticisms however as to whether Whole School Approaches engage sufficiently with schools as complex adaptive systems that require stakeholders to drive change (Turunen *et al.*, 2017; Bartelink *et al.*, 2019; Koh and Askell-Williams, 2021).

### The School Health and Wellbeing Improvement Research Network (SHINE)

To meet the need for data-driven approaches to inform practice in schools, SHINE was established in 2018 (https://shine.sphsu.gla.ac.uk/) as a pilot focusing on MHWB. SHINE aims to create a national infrastructure for school-based health improvement research and develop stronger links between research and educational communities in Scotland. Specifically, it aims to (i) build stronger research partnerships between schools, academic researchers and other important stakeholders, (ii) support its member schools to take a data-driven approach in addressing their HWB needs, with a particular focus on mental health and (iii) use data to facilitate a systems-level, Whole School Approach to health improvement. SHINE adopted a similar model to the School Health Research Network (SHRN) in Wales which is based on a research–policy–practice partnership with academic researchers working closely with the Welsh Government, Public Health Wales and member schools (Murphy *et al.*, 2021). SHRN is fully embedded in Wales and is focused mainly on secondary school children. In SHINE’s development phase, our team liaised with SHRN colleagues with continued engagement to identify best practice and learning. SHINE membership has grown substantially since this case study work began in October 2019 from 107 schools to 564 schools by June 2022, nearly a quarter of Scottish schools, representing 231 959 young people. Schools can join SHINE free of charge through signing up on the network’s website with approval from the school’s headteacher and the nomination of a school SHINE Lead.

SHINE aims to support schools to collect and interpret data from their pupils to create an evidence-base for school HWB planning. The Scottish Health Behaviour in School-aged Children (HBSC) survey underpinned SHINE’s pilot phase, with a longer-term aim of supporting schools to collect data from larger samples of their pupils. HBSC collects internationally comparable data every 4 years on 11-, 13- and 15-year-old’s health, health behaviours and their social context (http://www.hbsc.org/). Previously, HBSC-participating schools received a copy of the HBSC national report, but school-level data were not reported. During the SHINE pilot, school-level HBSC data were fed back to individual schools for the first time in the form of a school report in May 2019. The report presented results alongside a regional average. The HBSC sample was not designed to be representative at school level, therefore, the reports served as an exemplar of how schools might engage in a data-driven approach rather than providing a comprehensive picture of the HWB of pupils in the school. SHINE schools who did not participate in the 2018 Scottish HBSC survey (or had too few pupils to report at school level) received a regional level HWB report instead. The reports were provided to schools as a basis for planning HWB activities, informing curriculum development and engaging parents and pupils regarding health concerns and priorities. Topic areas included were MHWB, general health, sleep, electronic media use, bullying and discrimination, eating habits, physical activity, substance use, peer and family relationships, social support and the school environment. Figures were used to present data by gender and year group (primary 7, secondary years 2 and 4) where possible. Reports also included a section that signposted schools to further information and support from public sector and third sector organizations on the topics covered. Schools were not provided with a prescriptive way to approach the interpretation of the reports. It is therefore of interest to explore and understand how the reports were both received and applied within the school context.

### Aim

Through qualitative case studies, we aimed to explore how SHINE HWB data reports could be used to inform whole school action planning and facilitate collaborative approaches to health improvement, as well as identifying the opportunities and challenges in using research data to drive systems level change within the school setting. We were also interested in perceptions on participating in the SHINE network more widely during the pilot phase.

## METHOD

### Ethical issues

The University of Glasgow’s College of Medicine, Veterinary and Life Sciences Research Ethics Committee provided ethical approval for this project (UREC Reference: 200190008). We also obtained local education authority approval before approaching ­participants. We provided all participants with an information sheet and they signed a consent form. Pupils provided active written consent and parents provided opt-out consent.

### Study design

Using a case study design, we aimed to hear from a wide range of perspectives within schools, as well as understanding the perspectives of key LA actors to reflect a Whole School Approach.

### Recruitment

We identified four LAs from which to recruit participants and schools from attendees at a SHINE conference in May 2019 who indicated their willingness to participate in the case study research. From each we aimed to recruit a primary and secondary school, the LA HWB Lead and a Data Lead (staff with remit for HWB and data in the education department in the relevant local authorities). Characteristics of the eight schools are reported in Table 1.

**Table 1:** :—School characteristics

School ID	School type	School roll	Location	Denomination	% pupils in SIMD^a^ 1 & 2	% Eligible free school meals^b^	Senior leader	SHINE lead	Stakeholder	Staff focus group	Pupil focus group 1	Pupil focus group 2
A1	P	<100	Accessible rural area	Non-denominational	0%	40%	HT	X (DHT)	Class teacher	*n* = 5	P6/7 *n* = 5	
A2	S	<950	Other urban area	Roman Catholic	20 < 30%	11%	DHT	X	Listening service volunteer	*n* = 7	S2 *n* = 7	S3 *n* = 7
B1	S	<400	Accessible small towns	Non-denominational	0 < 10%	13%	HT	X	Youth Learning worker	*n* = 6	S2 *n* = 6	S4 *n* = 5
B2	P	<150	Other urban area	Non-denominational	10 < 20%	57%	HT	Not available	Inclusion & wellbeing	*n* = 6	S2 *n* = 6	S4 *n* = 6
C1	S	<1050	Other urban area	Non-denominational	20 < 30%	22%	DHT	X	not available	*n* = 6	S2 *n* = 4	S4 *n* = 7
C2	P	<400	Other urban area	Non-denominational	30 < 40%	77%	AHT	X (DHT)	Educational psychologist	*n* = 6	P7 *n* = 5	P7 *n* = 5
D1	S	<950	Large urban area	Non-denominational	10 < 20%	11%	DHT	X (PT)	Support for learning teacher	*n* = 3	P7 *n* = 6	P7 *n* = 4
D2	P	<350	Large urban areas	Roman Catholic	0 < 10%	40%	ADHT	X	Support for learning assistant	*n* = 5	S2 *n* = 4	S4 *n* = 4

School IDs A–D represent the four local authorities. PS, Primary School; S, Secondary School; HT, Headteacher; DHT, Deputy Headteacher; AHT, Acting Headteacher; ADHT, Acting Deputy Headteacher; PT, Principal Teacher; P6/7, Primary years 6/7; S2 S3 S4, Secondary years 2, 3, 4.

^a^SIMD is the Scottish Index of Multiple Deprivation (1 = the most deprived quintile).

^b^In Primary Schools this figure represents the % of the P4-7 school roll eligible for Free School Meals. All P1-3 children are eligible for Free School Meals in Scottish schools.

All but one school invited agreed to participate. Each SHINE member school has a staff member designated as the SHINE Lead who liaises with the SHINE team at the University of Glasgow. Through the SHINE Leads we invited a senior school leader, a relevant stakeholder (a non-teaching staff member who was predominantly involved in HWB within the school, e.g. school counsellor, educational psychologist) and the school SHINE Lead to participate in an interview. The SHINE Lead also assisted in the recruitment of participants for staff and pupil focus groups. We requested that they invite a cross-section of pupils, rather than only those who were most academic or enthusiastic to participate. The research team directly contacted LA HWB Leads and Data Leads within each of the four LAs.

### Study procedure

Interviews and focus groups were conducted from October 2019 to February 2020 in-person on school premises and LA offices, with a single interview conducted via telephone. All interviews and focus groups were recorded and transcribed verbatim. Topics discussed with school participants included: HWB priorities and activities in their school, engagement with the SHINE report, how the reports had/will/or could be used to inform school health improvement plans, and how SHINE fits with other school HWB activities and processes. We provided participants with a copy of either their school SHINE report or a regional report to aid the discussion. We provided pupils with a simplified pupil-friendly report (not previously distributed to schools) and excerpts from the main SHINE report for discussion. We asked LA participants about interaction from and with schools over the SHINE reports, ways in which the data had been used to help inform planning, and their LAs’ HWB data activities.

### Analysis

Transcripts were anonymized and allocated across four members of the research team D.H., J.M., J.B. and S.C. to read and re-read. The team discussed initial themes that were inductively generated creating a coding framework. These included: context, awareness of SHINE, response to reports, barriers to future use of SHINE reports, facilitators to using reports/engaging with SHINE, planning, pupils and parents, and the Scottish Government Health and Wellbeing Census. Each team member coded their allocated transcripts to the framework. Using the coded data, a process of deductive coding was applied using Normalisation Process Theory (NPT) as an overarching framework. NPT is a middle-range theory of implementation that can assist in identifying the extent to which an organization is working towards the full implementation of a new practice (May and Finch, 2009; May *et al.*, 2009). NPT is comprised of four components, each with their own sub-components. We followed McNaughton *et al.* (2020) in translating original NPT components; definitions for each are provided in Supplementary Table 1. We focused on the first three NPT components which cover the earlier stages of implementation as participants were not yet at an evaluative stage of their practice. The three higher-level components are: making sense of data-driven approaches; building a collaborative approach and undertaking a data-driven approach.

## RESULTS

### Participant information

Twenty-two school interviews and eight LA interviews were conducted, as well as eight school staff focus groups and 15 pupil focus groups. In total, 30 participants took part in an interview and 125 participants in a focus group. Interviews ranged from 18 to 58 minutes and focus groups from 20 to 42 min. In School, B2 we were unable to interview the SHINE Lead due to absence, and in School C1 no stakeholder was available for interview. One LA requested that an Educational Psychologist be interviewed in place of the HWB Lead.

### Making sense of a data-driven approach

#### Data-driven approaches as new practices

Participants described how they might best implement a data-driven approach to HWB action planning. All schools had numerous initiatives in place to support HWB, however, there did not always appear to be a clear evidence-based strategy for the use of data to select/evaluate interventions. Generally, participants were enthusiastic about the possibilities of using their own data to improve HWB provision in their school. Schools had collected pupil data on HWB previously and used it to inform decision-making, however, they usually did this with little expertise and support. Participants recognized the uniqueness of the SHINE model in providing schools with their own data, and the possibilities for SHINE to support schools and LAs with larger-scale data collection in future to drive action planning around HWB.

To have the research evaluative critical friend working alongside you as almost a mentor at strategic local authority level would be amazing.

(LA2 HWB Lead)

#### Individual interpretation

Compared with other participants, senior leaders and SHINE Leads were more advanced in considering the ways in which they could use a data-driven approach. In three LAs, participants also described the progress they had made in this area previously. One LA HBW Lead recognized the need to improve work on data use.

We know in [this Local Authority] that we do not use data well, and that’s something, again, in our Children’s Services Plan, that’s one of our priorities for a new plan.

(LA4 HWB Lead)

Participants in senior positions discussed the strategic benefits of using data, driven by the need to meet LA and national obligations around data use and reporting. SHINE Leads were often HWB Leads within their schools, and therefore, SHINE’s work fell naturally within their remit. The SHINE report was a resource to allow them to focus planning on the areas of pupils’ HWB that were identified as priorities. These staff recognized the benefits of having data to strengthen their rationales for decision-making, planning and funding, and some indicated that the SHINE report had already provided evidence to aid decision-making and support specific initiatives.

I think now I know that I can’t really make any decisions because I need the data, and this is ideal having the data.

(School A1, Headteacher)

Analysis indicated that stakeholders, other school staff and pupils had a less developed understanding of data-driven approaches to HWB planning and delivery. For many, this was their first opportunity to see the SHINE report and find out more about the SHINE network. Once they had seen the report, participants described the potential benefits a data-driven approach could bring within their schools and with pupils.

I think it would be a good tool for identifying future priorities, for example social media…our kids are way more comfortable sharing things online than the average was.

(School A2, Teacher Focus Group, Participant 1)

Quite good to see because then you can compare the different schools. And it shows the levels of confidence in people. It is a bit easier to help see what needs to be changed, how we need to look at things and what the schools need to address.

(School A1, Pupil Focus Group, Participant 4)

Some stakeholders and school staff, however, were unsure as to how they should interpret some of the findings. They questioned the extent to which pupils had answered the questions honestly and felt that the cross-sectional nature of data collection limited generalizability. There was also recognition that sample sizes in the reports were small, and therefore, limited in their ability to inform future action planning.

I presume possibly due to the small sample, but 100% of people feeling high in life satisfaction in second year. As a second year Personal and Social Development teacher I find that hard to believe.

(School A2, School Staff Focus Group, Participant 7)

#### Collective interpretation

Senior leaders and SHINE Leads described the potential of the data reports to enable a collective approach to HWB action planning among staff. This had already happened in some schools, and senior leaders and SHINE Leads recognized that a data-driven approach to HWB could complement their schools’ ethos through facilitating wider data interpretation by a range of school stakeholders to inform action planning.

Related to this was HWB as being the ‘responsibility of all’. This reflected the Scottish Government expectation that HWB is an integral component and cross-cutting theme which should be addressed by all the school staff across all areas of the Scottish curriculum (Scottish Government, no date).

It’s trying to ensure that all staff are on the same page around HWB and are confident enough and see that it is their role. They’re not a teacher of x, y and z, but it’s this holistic approach to it.

(School B1, Headteacher)

Sharing the results of school-level data was identified as a means through which to enable delivery of a Whole School Approach to HWB. A more communal approach across the full school community such as this had not yet been achieved in any schools. Nevertheless, the importance of including pupils in this process was recognized: data can give the pupils a voice leading to a greater communal understanding. This was raised by a pupil:

I think when you sometimes speak to adults they don’t always understand exactly how you’re feeling […]. And when you’re, like, feeling upset or something sometimes they don’t always understand. So it’s quite hard to tell them. But, like, if you see statistics and everything like that, you, kind of, see how much people are actually feeling that way.

(School A2, Pupil Focus Group, S3, Participant 3)

There was also recognition that the wider school community, including parents and pupils, needed to be engaged within a data-driven approach, which requires support. Pupils indicated that greater awareness of others’ feelings might develop greater empathy.

It’s knowing maybe what other people are going through a bit more and how you could maybe help them, if it’s not something you’re feeling, it may be something someone else is feeling.

(School D2, S4 Focus group, Participant 1)

When considering the wider school community, participants reflected on the communities in which their schools were embedded. Participants in the rural schools felt their pupils may be more protected from potentially damaging social issues experienced in urban schools. Conversely, participants in schools where more pupils were likely to be experiencing poverty, highlighted the need to manage the impact of challenging home lives experienced by pupils. In smaller schools, participants felt better able to identify pupils’ needs and to engage with their local communities. This was more challenging in Catholic schools where catchment areas were larger, and locating the school within a single community was not possible.

### Building a collaborative approach

#### Having the skills to engage

Senior school leaders, SHINE Leads and some LA participants recognized that taking a data-driven approach to HWB required additional data collection and analytical skills that were new to many school staff. To address this, LA HWB Leads described ways in which they tried to support school staff with data use and interpretation, for example, through building systems that allowed schools to collect data and analysis training for school HWB champions.

I think as an authority we provide our schools with a lot of data, we try to do some of the speed work for them. So we will try to create the kind of consistent systems to capture their data.

(LA2 HWB Lead)

Participants particularly valued the SHINE Team’s research expertise and access to support from SHINE.

People who are researchers, who can interpret the data more effectively than me...You can see the benefit of a skilled professional doing it.

(School A1, Headteacher)

Those who had been involved in data collection within schools previously valued the validated measures used within the HBSC survey, providing greater confidence in using the measures to collect data more extensively within their schools.

#### Organizing the school community

Senior school leaders and SHINE Leads expressed a desire to engage the whole school community in undertaking a data-driven approach to HWB and believed school-level reports were a means through which to achieve this.

You would have much more concrete evidence. And I think you would then have the staff on board with you more. Because I think that’s one of the keys - that we need to communicate with staff so they understand.

(School A2, SHINE Lead)

Nevertheless, at the time of interview, the extent to which the SHINE reports had been shared within schools varied. Most of the SHINE Leads and school senior leadership teams had seen the SHINE report but sharing beyond these groups was more limited. Although enthusiastic about sharing reports more widely, and engaging with others in planning in response to the reports, there was some caution at leadership level. The main concern was around staff, pupils and parents feeling overwhelmed, given the breadth of data included and the importance of understanding how to interpret it properly. There were suggestions that engagement should focus on specific areas of the report, be guided and contextualized by teachers for pupils, and carefully managed, for example, when speaking with parents.

I think some parents would find it useful. I think some parents would be quite confused with it…I think as well if there was too much data and it’s keeping it as clear as possible for the parent.

(School C1, Headteacher)

There were also concerns about how LAs might interpret data in terms of benchmarking, and how this might negatively impact schools.

#### Recognizing legitimate role

Most school staff and leaders recognized that pupil HWB was part of their role and were passionate about supporting their pupils. They reported greatest concern about a perceived rising prevalence of poor MHWB amongst pupils. However, some also felt that observed increases in MHWB issues may not always reflect a true deterioration in pupil mental health. For example, reductions in stigma may make it more likely that pupils report mental health concerns, greater awareness of issues such as anxiety may lead to more pupils self-diagnosing, and they felt that current cohorts of pupils may be less able to manage the emotions associated with normal adolescent development and experiences.

I think we’re naming things too early. Children who are maybe feeling a little bit uncomfortable, a little bit nervous which is normal, it’s just part of growing up, we’re giving it names.

(School D2, School Staff Focus Group, Participant 3)

It was also recognized that many issues exist beyond the school (e.g. social media), and therefore, a wider community approach is necessary for success in improving pupil HWB.

#### Defining sustainable actions

Participants were asked to consider actions needed to encourage the use of a data-driven approach to HWB action planning. Responses included receiving reports in the summer term ahead of planning for the new school year; more opportunities to learn from good practice through networking events and further support from SHINE that would help to reduce school staff workload. One Headteacher who had attended a SHINE national conference described the changes they had implemented following this event:

The thing that I really got out of coming to the conference was the Rights Respecting Schools and children’s rights. And we have a section within our learning journeys for health and wellbeing, and we came back and I’ve changed that.

(School A1 Headteacher)

An example of potential workload reduction was the enthusiasm with which staff described the signposting section of the SHINE reports. Participants commented that this provided trusted resources for them to plan activities with pupils, reducing substantial searching, sifting and critical assessment of external materials.

### Undertaking a data-driven approach

#### Making use of the SHINE reports

School senior leaders and staff with an assigned HWB role were beginning to embed information from the SHINE report into their planning. For example, in School A2, senior leaders directed staff to specific report sections on body image and this information formed the basis of activities within the school’s Wellbeing Week.

I don’t think any other staff have particularly seen the other bits but certainly that first bit is being used as part of the wellbeing group.

(School A2, Deputy Headteacher and SHINE Lead)

In terms of the report’s accessibility, responses were overwhelmingly positive. Participants noted the clear layout that was easy to navigate, graphs that were easy to interpret and information legible. They also welcomed the range of areas of HWB covered. Pupil participants were enthusiastic about interacting with the standard SHINE reports and preferred this report to the shorter ‘Pupil-Friendly’ version. They liked having access to all the information in graph format, rather than infographics, some of which they found difficult to understand.

I think [the main SHINE report] gives more information in a way that you wouldn’t have to try and work it out by yourself...I prefer the adult version just because it’s more there, and just exact.

(School A1, P6/7 Pupil Focus Group, Participant 1)

#### Working with and trusting the data-driven approach

As indicated above, those participants who were most aware of SHINE valued the Network’s expertise in data collection and analysis, as well as access to resources and examples of good practice provided. As discussed, there were some uncertainties about how to interpret the data due to small sample sizes. The HBSC sampling framework meant many schools only had data for one class in each year group. In working towards a robust whole-school approach to the use of HWB data, many schools expressed the wish to use validated measures with a wider cohort.

I think I’d like to have this information for more pupils if that would make sense, because I think I’m aware, you know, it was a relatively small cohort, but still because you would get a better overview

(School B1, HT)

Some school staff and other stakeholders also expressed a wish for individual pupil data, as well as anonymous aggregated data, in order to be able to follow-up with individual pupils if a need for support was identified. Smaller schools indicated that a report providing results for all primary schools in a secondary school cluster would be valuable in supporting primary–secondary transition.

#### Appropriate division of tasks

Despite enthusiasm from school participants to be involved in SHINE, and about the SHINE reports, only a small number of participants had read the full reports and undertaken action planning around their results. Senior staff and SHINE Leads highlighted staff workloads and reported a reluctance to overload school staff with additional tasks arising from the data reports. School senior leaders recognized this as part of their leadership role: prioritizing the issues to be addressed without creating too much additional demand on staff time.

We have got quite a lot of data now and its being mindful not to swamp staff and trying to support them…it’s been more just about taking one step at a time and making sure we’re in line with our priorities.

(School C2, Acting Headteacher)

Within this context, the SHINE Leads within schools had an important role as champions of SHINE and of using available data to inform health improvement activities within their schools. Despite the SHINE report only reporting data from one class, SHINE Leads could see the potential value of asking the same questions to a year-group or the whole school. Involvement with SHINE had provided the opportunity, expertise and support to enable schools to take forward a wider data-driven approach to HBW.

#### Allocating resources

Within the pilot phase, the new resources introduced were the SHINE reports. School senior leaders and SHINE Leads then had the responsibility for further dissemination of the report, and for action planning arising from it. These senior-level actors admitted that it was not only staff time that limited wider implementation, but also the limits on their own time.

We’ve so many priorities in the school, and even just the time taken up spending with kids which is what we’re here for and absolutely what I spend my time in school doing, but having this opportunity today and opening it up a wee bit I think will really help.

(School D2, SHINE Lead)

In smaller schools where staff levels were also smaller, there was further concern about sharing the load of additional work in relation to HWB and other responsibilities.

LA participants discussed the limitations of working with schools on school-level data, as they required LA level data and comparisons. Nevertheless, there were indications that LAs were supporting school staff through training and the provision of HWB programmes and resources, and that there was future potential for them to work with the SHINE network as a whole and with SHINE schools in their authority.

## DISCUSSION

This case study evaluation explored how SHINE HWB data reports could be used to inform whole school action planning and facilitate collaborative approaches to health improvement, and identify opportunities and challenges in using research data to drive systems level change within school settings. Results indicated that participants saw the SHINE reports and the additional support provided by SHINE as an opportunity to undertake data-driven approaches to HWB action planning. Specifically, senior leaders, school SHINE Leads and LAs found the data to be useful in identifying HWB needs and assisting with health improvement action planning. While many of the schools had carried out some data collection activities previously in response to national and LA commitments to embed data use in school improvement planning, there was a lack of consistency and confidence in their approach. SHINE’s expertise was an opportunity through which to overcome lack of knowledge and skills in relation to data collection, interpretation and use.

Given the early stage of introduction to the annual improvement cycle at the time of interview (just a few months), it is perhaps unsurprising that the SHINE reports had not been shared widely within the school community. This lack of sharing limited the opportunity for schools to use reports to inform action planning around HWB. Nevertheless, staff welcomed the prospect of engaging in professional dialogue around areas for development in this new way of working. Some specific issues were raised in relation to further development, including the management of staff workload, the best ways to share the results with pupils and parents and the support of SHINE to grow the staff skillset to interpret the results accurately and efficiently, particularly where small pupil numbers were a factor.

SHINE has implemented some of this feedback into its ongoing engagement with schools and LAs. Building from the pilot work described here, the network is developing schools’ and LAs’ capacity to collect, analyse and use data in school improvement planning. In response to mental health being identified as a key priority, and to promote a harmonized approach to measurement across the whole school, SHINE created a bespoke mental health survey available to all SHINE schools. Schools can administer the survey to pupils (aged 10–18 years) to complete online. A video has been developed for pupils explaining that they cannot be identified when completing the survey. Keeping in mind some participants’ concerns that anonymous data collection limits schools’ ability to help individual children, pupils are urged to speak with a trusted adult should completing the survey highlight areas where they would like further support. Data are then collated and analysed by the SHINE team and a report returned to schools within 2 weeks, providing a level of data expertise and presentation which schools do not usually have access to with HWB data. Schools can also distribute the survey to as many pupils as they wish, when they wish, allowing greater flexibility to maximize representation and ease of use. The data report follows a similar format to the previous SHINE school reports which were perceived as clear and accessible by both school staff and pupils. Additional guidance is provided in the reports on how to interpret measures used, as well as ‘challenge questions’ to promote discussion and interpretation of the data within their school community and a list of organizations to consult for additional support. It is hoped that schools are able to use this aggregated data alongside individual level data which was highlighted by other school stakeholders as being so important for supporting those pupils experiencing mental health difficulties.

SHINE is underpinned by a Whole School Approach that recognises schools as complex adaptive systems that constantly evolve and change. While the importance of a Whole School Approach is embedded within the Scottish education system (Scottish Government, 2021b, no date), in practice there is often a focus on short-term implementation rather than an evolving, sustainable process (Murphy *et al.*, 2021; Koh and Askell-Williams, 2021). Following Murphy *et al.* (2021), SHINE’s interactions with schools are conceptualized as a disruption to the existing school system, impacting school sub-systems including a range of agents (e.g. staff, pupils, parents, other relevant stakeholders) and components (e.g. HWB policies), as well as supra-systems (local education authority, wider community). Going forward, SHINE will continue to build partnerships with schools and LAs, monitor the impact of engagement in the SHINE network on the school system, and support schools in developing data-driven approaches to facilitate a collaborative approach to HWB improvement. It is also important to take into account the uniqueness of each school system and the ways in which interventions can be adapted to school contexts (Rosas, 2017; Bartelink *et al.*, 2019). Undoubtedly, as capacity and infrastructure building progresses within the SHINE network, its role will adapt and may focus more on supporting schools to co-produce and identify HWB interventions (disruptions) to the system that will best support their needs (Segrott and Roberts, 2019). There are only limited examples of similar initiatives elsewhere on which to base development of the network (Riley *et al.*, 2011; Murphy *et al.*, 2021), however, there are opportunities for innovative co-production activities with the school community to support a progressive and collaborative approach to HWB improvement. These approaches are in-line with the Scottish Government’s aim to increase schools’ engagement with parents and learner voice (Scottish Government, 2019).

### Strengths and limitations

A study strength was extensive data collection from a wide range of participants, including pupils, which revealed important differences in the role that these different actors played in data interpretation and action planning. Senior leaders and SHINE Leads were more likely to have engaged with their schools’ SHINE reports and considered ways that a data-driven approach could be taken forward in their schools. Other school stakeholders who were more likely to work with individual pupils, voiced some concerns about working with anonymized data in relation to following-up vulnerable young people.

In terms of the study sample, in recruiting participants, we selected LAs and schools that had indicated a willingness to participate in evaluation research. It is possible that we interviewed participants and schools who were more interested in using data for action planning and who were most enthusiastic about SHINE.

An additional strength was the use of the NPT framework to systematically identify the opportunities and challenges to a data-driven approach to school action planning, to understand where participants and schools were in the implementation process and identify developmental needs highlighted through professional dialogue. NPT can be a challenging framework to apply, therefore, we took time to translate concepts for the current study, whilst maintaining the original meanings and theoretical definitions. It was clear that schools were at a relatively early stage of implementation and were not yet at the point of appraising their use of data-driven approaches in schools to inform action planning. It will be important in future research to focus on how schools and LAs reflect on their involvement with SHINE and the extent to which they implement data-driven approaches to HWB action planning going forward.

## CONCLUSIONS

Participants recognized that data-driven approaches to HWB action planning strengthened rationales for decision-making, facilitated a collaborative approach to health improvement and fulfilled national-level expectations. Schools were enthusiastic about SHINE’s approach, the SHINE reports provided, and opportunities to access resources and expertise through the SHINE network. Schools were at an early stage of implementing data-driven approaches to HWB action planning based on the reports, and participants highlighted some important opportunities and challenges to implementation. SHINE has addressed some of the issues through enabling schools to collect MHWB data from a larger number of pupils. Collecting, analysing and utilizing data to inform and transform practice requires resources particularly in relation to staff time. It is therefore important that these initiatives clearly articulate the benefits and potential time and resource savings to schools, and that support is available for training and capacity building. Future work will continue to evaluate the impact of SHINE more widely in supporting schools to incorporate a data-driven approach into their HWB action planning.

## Supplementary Material

daac149_suppl_Supplementary_Material
