# Supplementary material for: Feasibility of using health and wellbeing data for school planning: the SHINE pilot in Scotland
Source: Health Promot Int. 2022 Nov 28;37(6):daac149. doi: 10.1093/heapro/daac149 (PMC9703801; doi:10.1093/heapro/daac149)
Supplement: daac149_suppl_Supplementary_Material [file daac149_suppl_supplementary_material.docx]

Supplementary Table 1 – Translated Normalisation Process Theory Components and Subcomponents

| **Making sense of a data-driven approach** | **Building a collaborative approach** | **Undertaking a data-driven approach** |
| --- | --- | --- |
| *Data-driven approaches as new practices*  Viewing data-driven approach as new way of working | *Having the skills to engage*    Work of actors leading policy implementation | *Making use of the SHINE reports*  Interactional work that enables/hinders tasks in school setting |
| *Individual interpretation*  An actor’s understanding of undertaking a data-driven approach | *Organising the school community*  (Re)organising others | *Working with and trusting the work of SHINE*  Confidence in data-driven approach and colleagues |
| *Communal Interpretation*  Work undertaken to reach shared understanding of data-driven approach | *Recognising legitimate role*  Work to ensure actors recognise their role in contributing to data-driven approach | *Appropriate division of tasks*  Allocating work to appropriately skilled staff as data-driven approach implemented |
| *Internalisation*  Perceived worth and benefits of engaging with data-driven approach (discussed across previous 3 subconcepts within data) | *Defining sustainable actions*  Understanding practices required to sustain data-driven approach | *Allocating resources*  Work shaped by resources and policies available |
